# Influence of the *PNPLA3* rs738409 Polymorphism on Non-Alcoholic Fatty Liver Disease and Renal Function among Normal Weight Subjects

**DOI:** 10.1371/journal.pone.0132640

**Published:** 2015-07-22

**Authors:** Kentaro Oniki, Junji Saruwatari, Tomoko Izuka, Ayami Kajiwara, Kazunori Morita, Misaki Sakata, Koji Otake, Yasuhiro Ogata, Kazuko Nakagawa

**Affiliations:** 1 Division of Pharmacology and Therapeutics, Graduate School of Pharmaceutical Sciences, Kumamoto University, Kumamoto, Japan; 2 Japanese Red Cross Kumamoto Health Care Center, Kumamoto, Japan; 3 Center for Clinical Pharmaceutical Sciences, Kumamoto University, Kumamoto, Japan; Institute of Medical Research A Lanari-IDIM, University of Buenos Aires-National Council of Scientific and Technological Research (CONICET), ARGENTINA

## Abstract

In normal weight subjects (body mass index < 25 kg/m^2^), non-alcoholic fatty liver disease (NAFLD) is likely to coexist with metabolic diseases. The *patatin-like phospholipase 3 (PNPLA3)* polymorphism rs738409 (c.444C>G) is associated with the risk of NAFLD and/or renal dysfunction; however, the influence of the weight status on the associations remains unknown. We aimed to clarify the associations of the *PNPLA3* polymorphism with the risk of NAFLD and/or renal dysfunction, while also paying careful attention to the weight status of the subjects. Cross-sectional and retrospective longitudinal studies with 5.5 ± 1.1 years of follow-up were conducted in 740 and 393 Japanese participants (61.2 ± 10.5 and 67.5 ± 6.0 years), respectively, during a health screening program. Among 591 subjects who did not have a habitual alcohol intake and/or hepatitis B or C virus infections, the *PNPLA3* G/G genotype was associated with the risk for NAFLD in normal weight subjects [odds ratio (95% CI): 3.06 (1.11–8.43), *P* < 0.05]. Among all subjects, carriers of the *PNPLA3* G/G genotype with a normal weight had a lower eGFR than those of the C/C genotype [partial regression coefficient (SE): -3.26 (1.48), *P* < 0.05]. These associations were replicated in the longitudinal analyses. Among the overweight subjects, none of the genotypes were significantly associated in the cross-sectional and longitudinal analyses; however, the power of the analyses was small, especially in the analyses among overweight subjects. The findings of this study suggest that carriers of the *PNPLA3* G/G genotype with a normal weight status should nevertheless be carefully monitored for the presence of NAFLD and/or renal dysfunction.

## Introduction

Non-alcoholic fatty liver disease (NAFLD) is a major health problem because the prevalence is increasing worldwide and the presence is an early predictor of future type 2 diabetes, cardiovascular disease (CVD) and chronic kidney disease (CKD) [1–3]. Obesity is strongly associated with the development and progression of NAFLD [1–4], however, NAFLD can also be observed in subjects with a normal weight status [body mass index (BMI) < 25 kg/m^2^] [5, 6]. Particularly in Asians, a high percentage of the patients with NAFLD have been found to have a normal weight status (15%–21%) and they appear to have different characteristics compared to overweight or obese patients (i.e., the differential distribution of visceral adipose tissue, recent increase in body weight and intake of high cholesterol diet and peculiar genetic background) [5]. A WHO expert consultation also suggested that Asians generally have a higher percentage of body fat than white people of the same age, sex, and BMI, and the proportion of Asian people with risk factors for type 2 diabetes and cardiovascular disease is substantial even below the existing WHO BMI cut-off point of 25 kg/m^2^ [7]. Feng et al. reported that normal weight subjects are more likely to have diabetes, hypertension and metabolic syndrome if they have NAFLD, thus suggesting that the normal weight individuals with NAFLD should be monitored more carefully than overweight or obese individuals with NAFLD [6].

The rs738409 polymorphism (encoding I148M) in the *patatin-like phospholipase 3* (*PNPLA3*) gene has been consistently recognized to be a major genetic factor for the development of NAFLD and advanced liver diseases, including steatohepatitis, cirrhosis and hepatocellular carcinoma [8–13]. PNPLA3 is expressed in the liver and adipose tissue and has acyl hydrolase activity [14]. The rs738409 polymorphism has been associated with the loss of the protein’s hydrolyzing function and with the hepatic triglyceride accumulation [14]. However, the effect of the subject weight status on the relationship between the *PNPLA3* polymorphism and the susceptibility to NAFLD remains unknown.

A recent meta-analysis demonstrated that both the presence and severity of NAFLD were associated with an increased development and severity of CKD [15]. Furthermore, experimental and epidemiological studies have suggested that NAFLD and CKD share common pathogenic mechanisms [16, 17]. Musso et al. recently suggested that the presence of the *PNPLA3* rs738409 polymorphism was associated with a lower estimated glomerular filtration rate (eGFR) and a higher prevalence of microalbuminuria and chronic kidney disease through a cross-sectional study [18], however, there is presently no data available regarding the longitudinal influence of *PNPLA3* polymorphism on the renal function, including the relationship to weight status.

The primary objective of this exploratory cross-sectional and longitudinal study is to investigate the relationships of the *PNPLA3* genotype with the risk for NAFLD and decline in the renal function among Japanese subjects, while also paying careful attention to the weight status of the subjects.

## Materials and Methods

### Subjects and study protocol

A cross-sectional case-control analysis was conducted among 740 subjects (478 males and 262 females, mean age: 61.2 ± 10.5 years) who were Japanese participants of the health screening program in the Japanese Red Cross Kumamoto Health Care Center between May 2003 and April 2012. Among these patients, a retrospective longitudinal analysis with 5.5 ± 1.1 [5.0 (1.0–6.0)] (mean ± SD [median (range)]) years of follow-up was performed in 393 subjects (238 males and 156 females, mean age at baseline: 67.5 ± 6.0 years) for whom longitudinal medical information could be collected between January 2006 and April 2012. The subjects with a habitual alcohol intake (consuming more than 30 g/day of alcohol in males and 20 g/day in females) and/or with positive serological tests for hepatitis B and C viruses were excluded according to the practical guidelines previously reported [19] on the analysis regarding the risk for NAFLD. This study protocol was approved by the ethics committees of the Faculty of Life Sciences, Kumamoto University and the Japanese Red Cross Kumamoto Health Care Center. All of the subjects provided their written informed consent prior to enrollment in the study.

### Measurements

The clinical information was recorded at each follow-up visit, i.e., at yearly intervals, although not all participants visited the center annually. The laboratory tests were performed using the standard methods of the Japan Society of Clinical Chemistry. The information regarding the alcohol intake and smoking habits was obtained via face-to-face interviews with health care providers. The BMI cut-off point (overweight) for health risk in different Asian populations varies from 22 kg/m^2^ to 25 kg/m^2^ [7]. Lowering of the cutoff values was indicated in Hong Kong Chinese, Indonesians, and Singaporeans, but not in northern Chinese and Japanese patients [7]. Therefore, a normal weight and overweight status were defined as BMI < 25 kg/m^2^ and BMI ≥ 25 kg/m^2^, respectively, in the present study. Hepatic ultrasonography scanning was used to diagnose fatty liver disease (FLD). FLD was diagnosed according to the following four criteria: a diffuse hyperechoic echotexture (bright liver), an increased echotexture in comparison to the kidneys, vascular blurring and deep attenuation [20]. The diagnosis of FLD was performed by the radiographer. A medical doctor then reviewed the images to evaluate the accuracy and reproducibility of the diagnosis. The FIB4 index is expected to be useful for evaluating hepatic fibrosis in NAFLD patients [21]. The values of the FIB4 index of the NAFLD subjects were calculated from the patients’ age, platelet counts, values of aspartate aminotransferase (AST) and alanine aminotransferase (ALT) using the following equation: [age × AST (IU/L)] / [(platelet count (10^9^) × √ALT (IU/L)], and the cut-off point of ≥ 2.67 for hepatic fibrosis was used [21]. The eGFR of each patient was calculated from the serum creatinine (SCr) level, age and gender using the following Japanese eGFR equation determined by the Japanese Society of Nephrology: eGFR (ml min^-1^ 1.73m^-2^) = 194×SCr (mg/dl) ^−1.094^× age^−0.287^(×0.739 if female) [22]. Diabetes was diagnosed based on the patients’ past history and the criteria recommended by the Expert Committee of the American Diabetes Association. Dyslipidemia was defined as a value of triglycerides (TG) ≥ 150 mg/dL, high-density lipoprotein cholesterol (HDL-C) < 40 mg/dL or low-density lipoprotein cholesterol (LDL-C) ≥ 140 mg/dL. Hypertension was defined as a systolic blood pressure (BP) ≥ 140 mmHg, a diastolic BP ≥ 90 mmHg or a history of hypertension.

### Genotyping

Genomic DNA was extracted from the whole blood using a DNA purification kit (FlexiGene DNA kit, QIAGEN, Hilden, Germany). The *PNPLA3* rs738409 polymorphism (c.444C>G, encoding I148M) was genotyped by a real-time TaqMan allelic discrimination assay (Applied Biosystems, CA, USA) according to the protocols provided by the manufacturers (assay no. C_7241_10). To ensure the genotyping quality, we included DNA samples as internal controls, hidden samples of a known genotype, and negative controls (water).

### Statistical analysis

The data are expressed as the means ± standard deviations, medians (range) for skewed variables or proportions for categorical variables. Categorical valuables were compared using Fisher’s exact test. Student’s *t*-test or a one-way ANOVA were used to compare the differences in the continuous parametric valuables. Nonparametric data were analyzed using the Mann-Whitney U test or Kruskal-Wallis test. The associations between the *PNPLA3* genotype and the risk of NAFLD or a decline in the eGFR were analyzed using bi-variable or multivariable logistic and linear regression models, respectively, according to the cross-sectional and longitudinal analyses. These associations were measured as the odds ratio (OR) and 95% confidence intervals (95% CI) for the risk of NAFLD, and the partial regression coefficient (Β) and standard error (SE) for the degree of fluctuation in the eGFR values. The ORs and B values of multivariable models were adjusted for potentially confounding factors, i.e., gender, diabetes, hypertension, dyslipidemia and fatty liver (only for the analysis of the eGFR values) as nominal variables, and age and BMI as continuous variables. In order to assess the effects of the weight status on the relationships of the *PNPLA3* genotype with the risk of NAFLD and decline in the eGFR, the study subjects were stratified by the presence of an overweight status at baseline. The interactive effects between the *PNPLA3* genotype and the presence of an overweight status on the risk of NAFLD and the eGFR values were additionally analyzed. On the longitudinal analyses, the generalized estimating equations approach was used to create the logistic and linear regression models. A value of *P* < 0.05 was considered to be statistically significant. The statistical power of the associations between the *PNPLA3* genotypes and the risk of NAFLD or the value of the eGFR were calculated at a significance (alpha) level of 0.05 (two-tailed) according to the sample size of this study using the SPSS Sample Power software program (version 2.0). All other statistical analyses were performed using the SPSS software package (version 17.0, IBM Japan Inc., Tokyo, Japan).

## Results

### Subjects characteristics

Among all 740 subjects, the frequencies of the *PNPLA3* C/C, C/G and G/G genotypes were 27.3%, 53.9% and 18.8%, respectively, and the frequencies of the genotype in the study subjects was consistent with previous reports in the Japanese population [23–25]. The observed genotype frequency distributions were consistent with the Hardy-Weinberg equilibrium (*P* > 0.05). The demographic characteristics of the subjects for the analyses regarding the risk for NAFLD are shown in Table 1. Among the subjects included in the cross-sectional analyses, the mean values of the BMI, waist circumstance, fasting blood glucose, diastolic BP, LDL-C, TG, AST, ALT, gamma-glutamyl transferase (GGT), the prevalence of being overweight, diabetes, dyslipidemia, ever-smoking status and the frequencies of *PNPLA3* C/G or G/G genotypes were significantly higher in the NAFLD subjects than in the non-NAFLD subjects, whereas the age, HDL-C and the frequency of females were significantly lower (Table 1). Among the subjects included in the longitudinal analyses, the mean values of the BMI, waist circumstance, fasting blood glucose, TG, AST, ALT, GGT, the prevalence of being overweight, diabetes and dyslipidemia were significantly higher in the NAFLD subjects than in the non-NAFLD subjects at baseline, whereas the value of HDL-C was significantly lower (Table 1). Among the NAFLD subjects in the cross-sectional and longitudinal analyses, the values of the FIB4 index were 1.49 (0.42–4.05), 1.58 (0.68–3.27), respectively, and the frequencies of an FIB4 index ≥ 2.67 were 7.6% and 3.9%, respectively (Table 1). In addition, the mean values of ALT in the NAFLD subjects were 32.0 ± 18.5 IU/L and 30.4 ± 16.5 IU/L, respectively (Table 1). The clinical characteristics of the subjects stratified by weight status are shown in S1 Table. The demographic characteristics did not differ between all 740 subjects and the 393 subjects, except for age (S1 Table). The mean age was greater in the 393 subjects (67.5 ± 6.0 years) than in all 740 subjects (61.2 ± 10.5 years). The clinical characteristics of the subjects for the cross-sectional analyses stratified by the weight status and the *PNPLA3* genotype are shown in S2 Table. Among the overweight subjects, the value of ALT was significantly higher in the *PNPLA3* C/G or G/G genotype than in those with the C/C genotype (S2 Table). The clinical characteristics of the 393 subjects stratified by the weight status and the *PNPLA3* genotype included in the longitudinal analyses at baseline are shown in S3 Table. Among the normal weight subjects, the value of GGT and the prevalence of NAFLD were significantly higher in the subjects with the *PNPLA3* C/G and/or G/G genotypes than in those with the C/C genotype (S3 Table). Since 149 subjects in the cross-sectional analysis and 52 subjects in the longitudinal analysis had habitual alcohol intake and/or were hepatitis B or C virus-positive, these subjects were excluded from the analyses regarding the risk of NAFLD. The demographic characteristics did not differ among the study subjects before and after excluding those exhibiting habitual alcohol intakes and/or a hepatitis B or C virus-positive status (data not shown).

**Table 1:** Clinical characteristics of the subjects for the analyses regarding the risk of NAFLD.

		Cross-sectional analysis			Longitudinal analysis (baseline)		
		Non-NAFLD	NAFLD	*P*	Non-NAFLD	NAFLD	*P*
		(N = 472)	(N = 119)		(N = 290)	(N = 51)	
Female (%) ^a^		205 (43.4)	36 (30.3)	< 0.01	122 (42.1)	23 (45.1)	0.759
Age (years)		65.2 ± 12.2	61.9 ± 11.4	< 0.01	67.9 ± 5.8	66.3 ± 5.8	0.064
BMI (kg/m^2^)		22.2 ± 2.7	25.6 ± 3.0	< 0.001	22.2 ± 2.6	25.0 ± 2.8	< 0.001
Waist circumstance (cm)		81.3 ± 7.9	89.7 ± 7.0	< 0.001	81.7 ± 7.7	87.9 ± 5.9	< 0.001
Fasting blood glucose (mg/dL) ^b^		95 (73–206)	100 (68–244)	< 0.001	96 (71–236)	105 (86–147)	< 0.001
Systolic BP (mmHg)		119.9 ± 15.9	122.7 ± 15.2	0.086	122.1 ± 18.1	126.2 ± 14.6	0.129
Diastolic BP (mmHg)		70.9 ± 10.1	74.8 ± 9.1	< 0.001	71.7 ± 10.6	73.1 ± 11.4	0.376
eGFR (ml/min/1.73m^2^)		72.4 ± 13.1	70.4 ± 13.9	0.158	72.4 ± 13.5	71.7 ± 14.3	0.717
LDL-C (mg/dL)		118.3 ± 26.0	132.1 ± 25.6	< 0.001	124.6 ± 26.4	130.8 ± 27.7	0.125
HDL-C (mg/dL)		70.2 ± 16.6	55.4 ± 12.8	< 0.001	71.4 ± 16.5	58.5 ± 13.2	< 0.001
TG (mg/dL) ^b^		83 (26–520)	131 (46–508)	< 0.001	87 (34–310)	121 (53–309)	< 0.001
AST (IU/L)		22.9 ± 5.5	27.1 ± 9.9	< 0.001	23.3 ± 5.6	27.4 ± 10.7	< 0.001
ALT (IU/L)		20.2 ± 8.4	32.0 ± 18.5	< 0.001	20.1 ± 6.7	30.4 ± 16.5	< 0.001
GGT (IU/L) ^b^		21 (6–302)	32 (8–180)	< 0.001	22 (7–132)	31 (9–259)	< 0.001
FIB4 index		-	1.49 (0.42–4.05)	-	-	1.58 (0.68–3.27)	-
FIB4 index ≥ 2.67		-	9 (7.6)	-	-	2 (3.9)	-
Overweight (%) ^a^		70 (14.8)	64 (53.8)	< 0.001	42 (14.5)	22 (43.1)	< 0.001
Diabetes (%) ^a^		51 (10.8)	28 (23.5)	< 0.01	28 (9.7)	15 (29.4)	< 0.001
Hypertension (%) ^a^		175 (37.1)	55 (46.2)	0.074	116 (40.0)	24 (47.1)	0.358
Dyslipidemia (%) ^a^		235 (49.8)	96 (80.7)	< 0.001	132 (45.5)	32 (62.7)	< 0.05
Ever smoking (%) ^a^		159 (33.7)	53 (44.5)	< 0.05	95 (32.8)	19 (37.3)	0.524
*PNPLA3* ^a^	C/C	143 (30.3)	23 (19.3)	< 0.05	86 (29.7)	9 (17.6)	0.150
	C/G	249 (52.8)	69 (58.0)		153 (52.8)	34 (66.7)	
	G/G	80 (16.9)	27 (22.7)		51 (17.6)	8 (15.7)	

The data are the means ± standard deviation, median (range) for skewed variables, or the numbers of subjects (%) for categorical variables.

^a^ Fisher’s exact test.

^b^ Mann–Whitney U test (otherwise, Student’s t-test was used).

NAFLD, non-alcoholic fatty liver disease; BMI, body mass index; BP, blood pressure; eGFR, estimated glomerular filtration rate; LDL-C, low-density lipoprotein cholesterol; HDL-C, high-density lipoprotein cholesterol; TG, triglyceride; AST, aspartate aminotransferase; ALT, alanine aminotransferase; GGT, gamma-glutamyl transferase; PNPLA3, patatin-like phospholipase 3.

### The effects of the *PNPLA3* genotype and weight status on the risk for NAFLD

Among the 591 subjects included in the cross-sectional multivariable logistic regression analyses of NAFLD, the risk of NAFLD was significantly higher in the subjects with the *PNPLA3* C/G or G/G genotypes than in those with the C/C genotype [adjusted OR (95% CI): 2.31 (1.26–4.25) or 2.83 (1.31–6.11), respectively] as well as in the normal weight subjects with the *PNPLA3* G/G genotype [adjusted OR (95% CI): 3.06 (1.11–8.43)], whereas the genotypes were not found to show significant associations in the overweight subjects (Table 2). There were no statistically significant interactions between the *PNPLA3* genotype and the weight status on the risk of NAFLD (*P* = 0.58).

**Table 2:** The effect of the *PNPLA3* genotype on the risk of NAFLD identified in the cross-sectional and longitudinal multivariable logistic regression analyses.

		All		Normal weight		Overweight	
		NAFLD / Non-NAFLD^a^	OR (95% CI) ^b^	NAFLD / Non-NAFLD^a^	OR (95% CI) ^b^	NAFLD / Non-NAFLD^a^	OR (95% CI) ^b^
Cross-sectional analysis							
*PNPLA3*	C/C	23 / 143	1	9 / 116	1	14 / 27	1
	C/G	69 / 249	2.31 (1.26–4.25)	34 / 215	2.20 (0.96–5.00)	35 / 34	2.30 (0.93–5.69)
	G/G	27 / 80	2.83 (1.31–6.11)	12 / 71	3.06 (1.11–8.43)	15 / 9	3.01 (0.92–9.89)
Age		-	0.98 (0.96–1.00)	-	0.99 (0.96–1.02)	-	0.97 (0.94–1.01)
Gender	Male	83 / 267	1	36 / 223	1	47 / 44	1
	Female	36 / 205	0.61 (0.36–1.03)	19 / 179	0.85 (0.44–1.64)	17 / 26	0.44 (0.18–1.06)
BMI		-	1.50 (1.35–1.66)	-	1.75 (1.39–2.21)	-	1.33 (1.08–1.64)
Diabetes	Absent	91 / 421	1	44 / 362	1	47 / 59	1
	Present	28 / 51	1.92 (1.04–3.55)	11 / 40	1.90 (0.84–4.30)	17 / 11	2.03 (0.78–5.26)
Hypertension	Absent	64 / 297	1	32 / 262	1	32 / 35	1
	Present	55 / 175	1.03 (0.62–1.72)	23 / 140	1.07 (0.56–2.05)	32 / 35	1.05 (0.44–2.48)
Dyslipidemia	Absent	23 / 237	1	8 / 207	1	15 / 30	1
	Present	96 / 235	3.63 (2.06–6.37)	47 195	4.83 (2.13–10.94)	49 / 40	2.42 (1.01–5.82)
Longitudinal analysis							
*PNPLA3*	C/C	15 / 80	1	7 / 68	1	8 / 12	1
	C/G	42 / 145	2.67 (1.30–5.49)	28 / 129	4.01 (1.34–12.02)	14 / 16	1.73 (0.68–4.38)
	G/G	18 / 41	3.09 (1.26–7.58)	11 / 34	5.47 (1.64–18.23)	7 / 7	1.78 (0.55–5.78)
Age		-	0.98 (0.93–1.03)	-	0.97 (0.91–1.04)	-	0.98 (0.92–1.05)
Gender	Male	48 / 148	1	27 /128	1	21 / 20	1
	Female	27 / 118	1.28 (0.66–2.47)	19 / 103	2.00 (0.95–4.23)	8 / 15	0.77 (0.28–2.14)
BMI		-	1.47 (1.32–1.65)	-	1.99 (1.59–2.51)	-	1.27 (1.06–1.52)
Diabetes	Absent	50 / 225	1	36 / 197	1	14 / 28	1
	Present	25 / 41	1.86 (0.91–3.79)	10 / 34	2.81 (1.14–6.94)	15 / 7	1.27 (0.53–3.05)
Hypertension	Absent	34 / 129	1	24 / 123	1	10 / 6	1
	Present	41 / 137	0.78 (0.44–1.39)	22 / 108	1.11 (0.59–2.08)	19 / 29	0.41 (0.18–0.94)
Dyslipidemia	Absent	22 / 126	1	12 / 111	1	10 / 15	1
	Present	53 / 140	1.96 (1.16–3.31)	34 / 120	1.59 (0.82–3.09)	19 / 20	2.79 (1.32–5.93)

^a^ In the longitudinal analysis, the numbers of NAFLD subjects and non-NAFLD subjects at the endpoint are shown.

^b^ Adjusted by all covariates.

PNPLA3, patatin-like phospholipase 3; NAFLD, non-alcoholic fatty liver disease; OR, odds ratio; CI, confidence interval; BMI, body mass index

**Fig 1:**
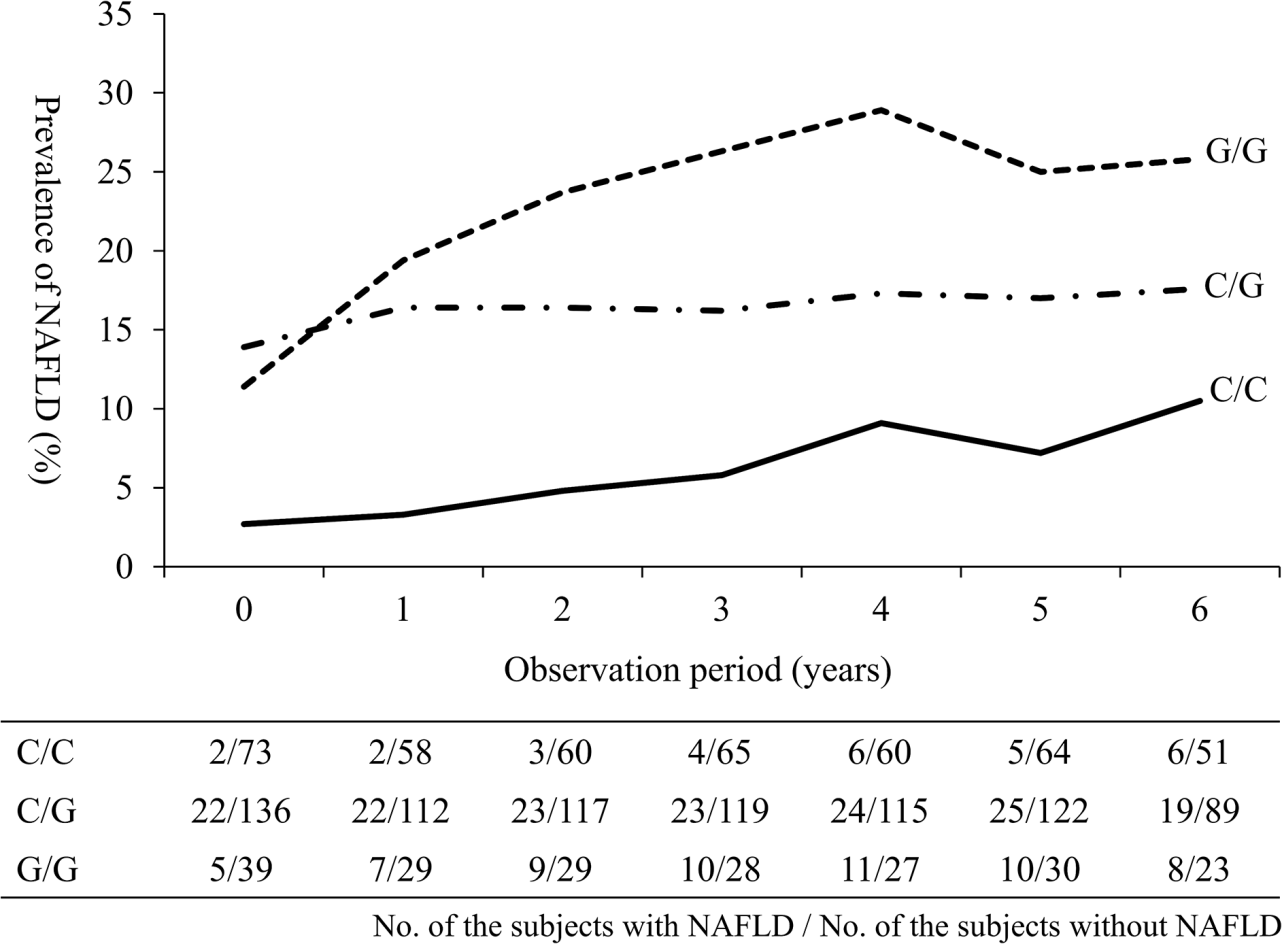
The longitudinal changes in the prevalence of NAFLD stratified by the *PNPLA3* genotype among normal weight subjects. The prevalence of NAFLD is shown as solid, dashed-dotted and dotted lines in the subjects with the *PNPLA3* C/C, C/G and G/G genotypes, respectively. NAFLD, non-alcoholic fatty liver disease; PNPLA3, patatin-like phospholipase 3.

To verify the results observed in the cross-sectional analyses, we also performed longitudinal multivariable logistic regression analyses among the 341 subjects. The prevalence of NAFLD at baseline and at the endpoint was 15.0% and 22.0%, respectively. Table 2 shows the longitudinal association between the *PNPLA3* genotype and the risk for NAFLD among the 341 subjects, both 277 normal weight and 64 overweight subjects, respectively. Among the 341 subjects, the longitudinal risk for NAFLD was significantly higher in the subjects with the *PNPLA3* C/G or G/G genotypes than in those with the C/C genotype [adjusted OR (95% CI): 2.67 (1.30–5.49) or 3.09 (1.26–7.58), respectively], and further increased the risk among the normal weight subjects with the *PNPLA3* C/G or G/G genotypes [adjusted OR (95% CI): 4.01 (1.34–12.02) or 5.47 (1.64–18.23), respectively] (Table 2). In addition, the prevalence of NAFLD was higher in the *PNPLA3* G/G carriers with normal weight during the observation period (Fig 1). Conversely, the *PNPLA3* genotype was not significantly associated with the longitudinal risk for NAFLD among the overweight subjects (Table 2). The cross-sectional and longitudinal bi-variable logistic regression models are shown in the S4 Table.

### The effects of weight status on the association between the *PNPLA3* genotype and the eGFR

In cross-sectional multiple linear regression analyses, no association was observed between the *PNPLA3* genotype and the eGFR values among all 740 subjects (Table 3). Conversely, the eGFR values were significantly lower in the subjects with the *PNPLA3* G/G genotype than in those with the C/C genotype among the 563 normal weight subjects [adjusted B (SE): -3.26 (1.48), *P* < 0.05], but the association was not observed among the 177 overweight subjects (Table 3). A significant interactive effect was observed between the *PNPLA3* genotype and the weight status on the eGFR values among all 740 subjects (*P* = 0.007).

**Table 3:** The effect of the *PNPLA3* genotype on the values of eGFR identified in the cross-sectional and longitudinal multiple linear regression analyses.

		All				Normal weight				Overweight			
		N	B^a^	SE	*P*	N	B ^a^	SE	*P*	N	B ^a^	SE	*P*
Cross-sectional analysis													
*PNPLA3*	C/C	202	0			149	0			53	0		
	C/G	399	-0.48	1.03	0.640	305	-0.94	1.18	0.423	94	1.29	2.08	0.535
	G/G	139	-1.63	1.31	0.214	109	-3.26	1.48	< 0.05	30	2.62	2.81	0.354
Age		-	-0.41	0.04	< 0.001	-	-0.41	0.04	< 0.001	-	-0.39	0.08	< 0.001
Gender	Male	478	0			349	0			129	0		
	Female	262	4.85	0.93	< 0.001	214	4.04	1.05	< 0.001	48	6.44	2.12	< 0.01
BMI		-	-0.18	0.17	0.284	-	-0.34	0.26	0.206	-	0.62	0.47	0.190
Diabetes	Absent	631	0			496	0			135	0		
	Present	109	0.29	1.26	0.821	67	1.00	1.55	0.518	42	-0.58	2.23	0.796
Hypertension	Absent	446	0			360	0			86	0		
	Present	294	-2.77	0.95	< 0.01	203	-1.74	1.09	0.110	91	-5.43	2.01	< 0.01
Dyslipidemia	Absent	338	0			276	0			62	0		
	Present	402	-2.50	0.92	< 0.01	287	-3.10	1.05	< 0.01	115	-0.34	2.05	0.868
FLD	Absent	590	0			496	0			94	0		
	Present	150	-1.27	1.24	0.306	67	-1.20	1.62	0.461	83	-1.99	1.99	0.319
Longitudinal analysis													
*PNPLA3*	C/C	107	0			85	0			22	0		
	C/G	216	-0.51	1.31	0.699	174	-1.69	1.49	0.255	42	3.47	2.59	0.180
	G/G	70	-2.74	1.70	0.107	55	-3.96	1.95	< 0.05	15	0.66	2.95	0.822
Age		-	-0.38	0.09	< 0.001	-	-0.40	0.10	< 0.001	-	-0.35	0.16	< 0.05
Gender	Male	237	0			183	0			54	0		
	Female	156	4.21	1.26	< 0.01	131	4.37	1.41	< 0.01	25	3.43	2.93	0.241
BMI		-	-0.13	0.21	0.535	-	-0.10	0.33	0.768	-	0.41	0.49	0.398
Diabetes	Absent	342	0			280	0			62	0		
	Present	51	2.52	1.46	0.084	34	1.32	1.80	0.463	17	4.69	2.15	< 0.05
Hypertension	Absent	228	0			198	0			30	0		
	Present	165	-1.92	1.10	0.081	116	-1.25	1.21	0.303	49	-3.65	2.20	0.098
Dyslipidemia	Absent	211	0			176	0			35	0		
	Present	182	-2.87	1.01	< 0.01	138	-2.98	1.18	< 0.05	44	-2.06	2.20	0.349
FLD	Absent	330	0			281	0			49	0		
	Present	63	-2.99	1.52	0.050	33	-1.43	2.02	0.481	30	-5.97	2.21	< 0.01

^a^ Adjusted by all covariates.

PNPLA3, patatin-like phospholipase 3; eGFR, estimated glomerular filtration rate; B, partial regression coefficient; SE, standard error; BMI, body mass index; FLD, fatty liver disease.

**Fig 2:**
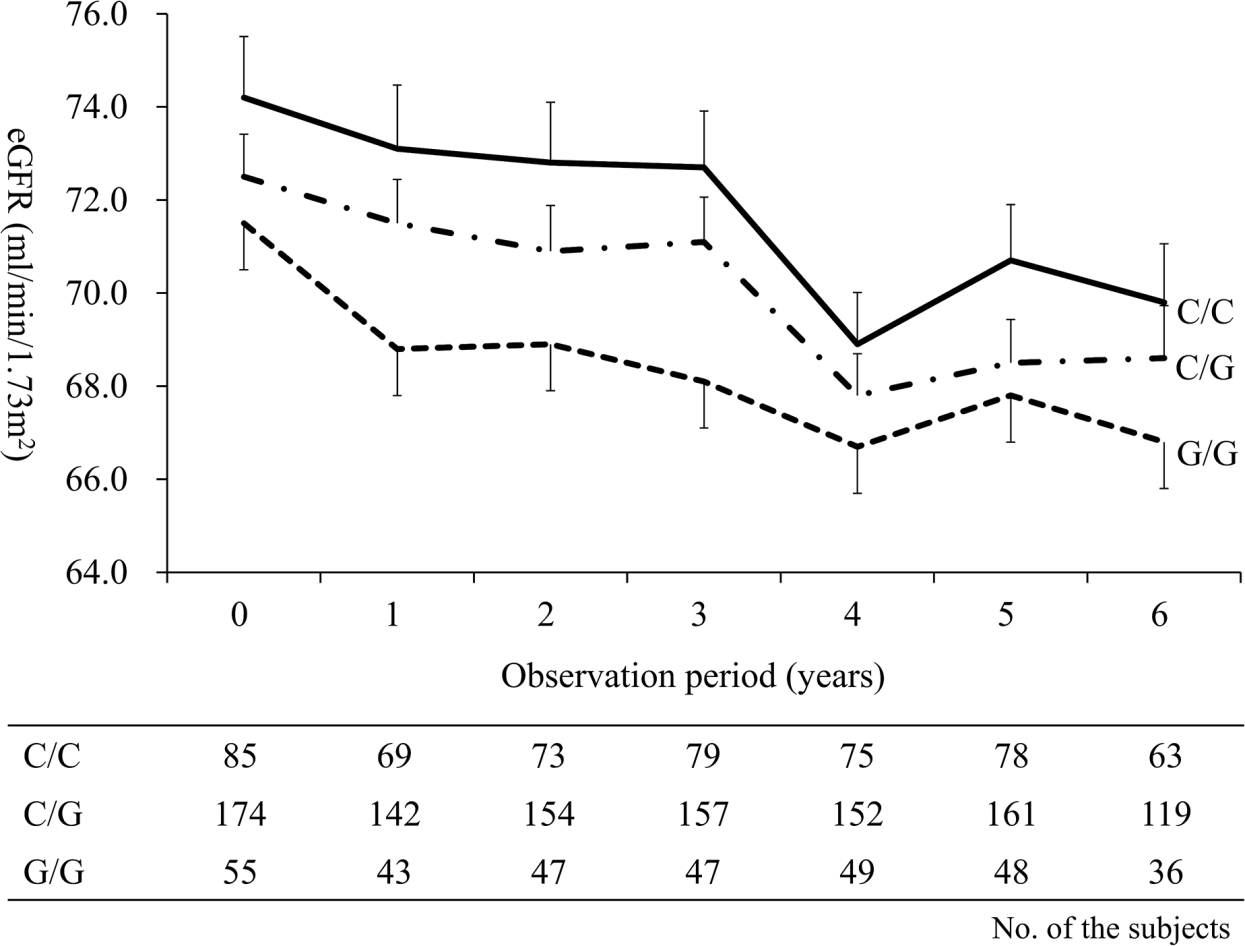
The longitudinal changes in eGFR stratified by the *PNPLA3* genotype among normal weight subjects. The mean values of eGFR are shown as solid, dashed-dotted and dotted lines in the subjects with the *PNPLA3* C/C, C/G and G/G genotypes, respectively, and the SEs are shown as antennae. eGFR, estimated glomerular filtration rate; PNPLA3, patatin-like phospholipase 3; SE, standard error.

We also performed longitudinal multiple linear regression analyses among the 393 subjects in order to verify the results observed in the cross-sectional analyses. The longitudinal effects of the *PNPLA3* genotype on the eGFR values are shown in Table 3. The eGFR values tended to be lower in the subjects with the *PNPLA3* G/G genotype than in those with the C/C genotype, however, this association was not statistically significant (Table 3). Conversely, the eGFR values were significantly lower in the subjects with the *PNPLA3* G/G genotype than in those with the C/C genotype among the 314 normal weight subjects [adjusted B (SE): -3.96 (1.95), *P* < 0.05] (Table 3). The *PNPLA3* G/G carriers with normal weight status had lower eGFR values throughout the observation period (Fig 2). No association was observed between the *PNPLA3* genotype and the eGFR value among the 79 overweight subjects (Table 3). In order to assess the effects of the presence of NAFLD on the association between the *PNPLA3* genotype and decline in eGFR, we also analyzed the effect of the *PNPLA3* genotype on the longitudinal eGFR values among the non-NAFLD and NAFLD subjects. The eGFR values were significantly lower in the subjects with the *PNPLA3* G/G genotype than in those with the C/C genotype among the non-NAFLD subjects [adjusted B (SE): -5.55 (1.96), *P* < 0.01], although no such association was observed between the *PNPLA3* genotype and the eGFR values among the NAFLD subjects [adjusted B (SE): 2.40 (4.23), *P* = 0.57]. The cross-sectional and longitudinal bi-variable linear regression models are shown in S5 Table.

### Statistical power analyses

In the cross-sectional analyses, the statistical power of the association analyses between the *PNPLA3* genotype and the risk for NAFLD or the value of the eGFR was 87% or 87% for all subjects, 61% or 71% for normal weight subjects and 43% or 26% for overweight subjects, respectively. The statistical power in the longitudinal analyses was 70% or 54% for all subjects, 40% or 40% for normal weight subjects and 22% or 13% for overweight subjects, respectively.

## Discussion

To the best of our knowledge, this is the first report to demonstrate that the effects of the *PNPLA3* rs738409 polymorphism on the risk for NAFLD and decline in eGFR were significant in normal weight subjects through the cross-sectional and longitudinal analyses. In Asians, the prevalence of NAFLD is equivalent to that in Caucasians, despite having lower BMI [4–6]. Asians with NAFLD have been known to exhibit a predominantly impaired insulin secretion [5]. Among the normal weight subjects, the presence of NAFLD was more strongly associated with the prevalence of diabetes, hypertension and metabolic syndrome compared to the obese/overweight subjects [6]. Therefore, clarifying the risk factors for NAFLD among the normal weight subjects will help to identify susceptible populations for the early prevention and treatment of NAFLD and its complications, especially in Asians. The findings of this study suggest that the *PNPLA3* rs738409 polymorphism may be utilized for the early detection of the high-risk group for NAFLD and decline in the renal function, even though individuals may have a normal weight status.

Although the *PNPLA3* rs738409 polymorphism is the only gene that has been consistently confirmed to be associated with the risk for NAFLD by genome-wide association studies and candidate gene studies [8–13], the exact mechanism underlying the association between the *PNPLA3* polymorphism and the incidence for NAFLD is a matter of debate. PNPLA3 is highly expressed in the human liver and adipose tissue [26] and plays a role in the hydrolysis of three major glycerolipids (i.e., triacylglycerol, diacylglycerol, and monoacylglycerol); the rs738409 G allele results in a loss of function impairing glycerolipids hydrolysis [14, 27]. In non-obese subjects, elevated ALT and TG levels, a higher degree of insulin resistance, increased waist circumstance, body weight change and an age between 40 and 64 years were identified as the risk factors for NAFLD [5, 28]. Hyysalo et al. previously examined the effects of NAFLD on the circulating lipid signature in relation to either obesity or the *PNPLA3* polymorphism [29]. Obesity-related NAFLD was associated with multiple changes in triacylglycerols, which may be attributed to obesity and/or insulin resistance rather than increased liver fat content per se [29]. The *PNPLA3*-related NAFLD was characterized by absolute and relative deficiencies of circulating triacylglycerols compared to obesity-related NAFLD, thus suggesting that the *PNPLA3* polymorphism may impair lipolysis rather than stimulate the synthesis of intrahepatocellular triacylglycerols [29]. Meanwhile, Shen et al. reported that the *PNPLA3* rs738409 G allele increased the risk for NAFLD, especially in the subjects without metabolic syndrome [12]. According to the findings of the present study and these previous studies, we speculate that the relationship between the *PNPLA3* polymorphism and the impaired lipolysis may be more pronounced in the normal weight subjects than in overweight subjects, thus resulting in the increased risk for NAFLD among the normal weight subjects with the *PNPLA3* rs738409 G/G genotype.

In the present study, the *PNPLA3* G/G genotype was associated with a longitudinal decline in the renal function only among normal weight subjects (Table 3 and Fig 2). The exact mechanisms underlying the association between *PNPLA3* rs738409 polymorphism and the decline in the renal function are currently unclear. Since NAFLD is closely related with the risk for CKD [15–17], *PNPLA3* rs738409 polymorphism may therefore be associated with a decline in the renal function through the presence and/or progression of NAFLD. On the other hand, we showed that the *PNPLA3* G/G genotype was also associated with a lower eGFR in the subjects without NAFLD during the observation period. A recent cross-sectional study indicated that the *PNPLA3* rs738409 G allele was associated with a lower eGFR and with a higher prevalence of microalbuminuria and CKD in 202 non-obese non-diabetic subjects regardless of the presence of NAFLD [18]. An extensive analysis of the *PNPLA3* mRNA expression in human tissues showed a high expression in the retina and sinusoidal pericytes (the hepatic stellate cells) [30]. In addition, PNPLA3 demonstrated a retinyl-esterase activity *ex vivo* and *in vitro*, which was lost as a result of the *PNPLA3* rs738409 polymorphism [30]. Romeo et al. speculated that the *PNPLA3* G allele may be associated with kidney disease due to an unbalanced activation of the kidney pericytes (i.e., glomerular podocytes) which play a pivotal role in the regulation of glomerular filtration [31]. Therefore, the *PNPLA3* rs738409 polymorphism may also directly impact the decline in renal function.

The *PNPLA3* G/G genotype was associated with not only the risk for NAFLD [8–12], but also a higher severity of carotid atherosclerosis in NAFLD patients [32]. Recently, Shen et al. investigated the effects of the *PNPLA3* genotype on the response to a lifestyle modification program based on a strategy for increasing energy expenditure and reducing the caloric intake for 12 months in non-diabetic NAFLD patients by a post-hoc analysis of a randomized controlled trial [33]. Interestingly, the *PNPLA3* G/G genotype carriers were more sensitive to the beneficial effects of lifestyle modification (i.e., the reduction in TG, total cholesterol, LDL-C, body weight and the waist to hip ratio), despite the G/G genotype carriers had a higher risk for NAFLD [33]. Meanwhile, it is well known that NAFLD and/or CKD patients have a strong risk of diabetes, hypertension, dyslipidemia, end-stage renal disease (ESRD) and CVD events [34]. The evidence obtained from the meta-analysis suggested that the early decline in proteinuria induced by the treatment was associated with a lower risk of elevation of the SCr level, ESRD or death [35]. The results of this study suggest that determining the *PNPLA3* genotype may be useful for preventing and treating NAFLD as well as a decline in the eGFR and associated complications, especially in normal weight individuals, by means of implementing targeted prevention and treatment programs for *PNPLA3* G/G genotype carriers.

Among the overweight subjects in this study, we observed no significant associations between the effects of the *PNPLA3* genotype and the risk of NAFLD and/or a decline in eGFR (Tables 2 and 3). Obesity is strongly associated with the development and progression of NAFLD and CKD due to insulin resistance, diabetes, hypertension, altered adipokine levels and/or glomerular hypertrophy [1–4, 36]. In the present study, the overweight subjects also had a high prevalence of NAFLD, lower eGFR values and more metabolic risk factors associated with the risk of NAFLD and a decline in the renal function (i.e., high prevalence of diabetes, hypertension, dyslipidemia and FLD) than the normal weight subjects (Table 1 and S2 Table). These metabolic risk factors may more strongly affect the development of NAFLD and/or decline in the renal function than the *PNPLA3* genotype in overweight subjects. On the other hand, previous reports have shown that obesity is one of the triggers of liver damage in the carriers of the *PNPLA3* G/G genotype [27]. Among the overweight subjects included in the cross-sectional study, the *PNPLA3* G/G genotype was significantly associated with an increased ALT value and tended to exhibit a higher prevalence of NAFLD (S2 Table and Table 2). The present study had a small sample size, and particularly in the analyses of the overweight subjects, the values of statistical power was below the necessary limit of power (i.e., 80%) to predict the development of NAFLD and the decline of the eGFR. Therefore, this study did not have sufficient power to clarify whether the *PNPLA3* polymorphism is an independent risk factor for NAFLD and/or a decline in the eGFR in overweight subjects, and a type 2 error cannot be excluded.

In the longitudinal analyses, the prevalence of NAFLD was higher in normal weight subjects with the *PNPLA3* C/G genotype in comparison to the G/G genotype at baseline (Fig 1), although this association was not observed in the cross-sectional analyses. At baseline, the *PNPLA3* C/G genotype carriers tended to have hypertension or dyslipidemia compared to the G/G genotype carriers, and therefore these risk factors may be associated with the high prevalence of NAFLD. However, the associations did not reach statistical significance and the underlying mechanism remains unclear. The study subjects were participants of the Japanese health screening program and might have had a high level of health literacy. In addition, the study subjects included in the longitudinal analyses were comparatively old (mean age: 67.5 ± 6.0 years at baseline and 72.9 ± 6.1 at endpoint). Therefore, a selection bias may be associated with the present study.

Other limitations of the present study should be noted. We included Japanese subjects, however, the ethnicity of the patients was self-reported in face-to-face interviews and not checked (e.g., by determining the birth places of the grandparents). The subjects’ alcohol consumption was evaluated through face-to-face interviews, which may have lacked reliability. Hepatic ultrasonography scanning, which is known to have a sensitivity of 64% and a specificity of 97% in detecting fatty liver [37], was used to diagnose FLD. The diagnosis was not confirmed by liver biopsy. Among the NAFLD subjects, the values of the FIB4 index and ALT were relatively low (Table 1). Therefore, the severity of the NAFLD subjects was relatively low, and most of the NAFLD subjects were thought to have simple steatosis. The present study is retrospective design and the study subjects were volunteers in a healthy screening program, which may have created a selection bias (e.g., there is possibility that overweight subjects may have moved from health screening to the treatment of obesity-related diseases and thus the effects of *PNPLA3* genotype might have only been observed among normal weight subjects).

In conclusion, this study provided preliminary findings demonstrating that the *PNPLA3* rs738409 genotypes are associated with the risk of NAFLD and decline of the eGFR in normal weight subjects, thus suggesting that even though carriers of the *PNPLA3* G/G genotype may have a normal weight status, they should nevertheless be carefully monitored for the presence of NAFLD, a decline in the renal function and associated complications. The information regarding the *PNPLA3* genotype in normal weight subjects may be utilized for health promotion, although further investigations with a larger number of subjects are needed to verify and further clarify these findings.

## Supporting Information

S1 Table Clinical characteristics of the subjects stratified by weight status. (DOCX)

S2 Table Clinical characteristics of all 740 subjects stratified by weight status and the *PNPLA3* genotype. (DOCX)

S3 Table Clinical characteristics at baseline of 393 subjects included in the longitudinal analyses stratified by the weight status and the *PNPLA3* genotype. (DOCX)

S4 Table The effect of the *PNPLA3* genotype on the risk of NAFLD identified in the cross-sectional and longitudinal bi-variable logistic regression analyses. (DOCX)

S5 Table The effect of the *PNPLA3* genotype on the values of eGFR identified in the cross-sectional and longitudinal bi-variable linear regression analyses. (DOCX)
