# Supplementary material for: Influence of the PNPLA3 rs738409 Polymorphism on Non-Alcoholic Fatty Liver Disease and Renal Function among Normal Weight Subjects
Source: PLoS One. 2015 Jul 22;10(7):e0132640. doi: 10.1371/journal.pone.0132640 (PMC4511733; doi:10.1371/journal.pone.0132640)
Supplement: S1 Table — (DOCX) [file pone.0132640.s001.docx]

**S1 Table. Clinical characteristics of the subjects stratified by weight status.**

|  | | Cross-sectional analysis | | |  | Longitudinal analysis (at baseline) | | |
| --- | --- | --- | --- | --- | --- | --- | --- | --- |
|  | | Normal weight  (N = 563) | Overweight  (N = 177) | *P* |  | Normal weight  (N = 314) | Overweight  (N = 79) | *P* |
| Female (%) ^a^ | | 214 (38.0) | 48 (27.1) | < 0.01 |  | 131 (41.7) | 25 (31.6) | 0.123 |
| Age (years) | | 61.6 ± 10.4 | 59.6 ± 10.8 | < 0.05 |  | 67.5 ± 5.8 | 67.3 ± 6.4 | 0.757 |
| BMI (kg/m^2^) | | 21.8 ± 2.0 | 26.9 ± 2.1 | < 0.01 |  | 21.7 ± 2.0 | 26.7 ± 2.0 | < 0.001 |
| Waist circumstance (cm) | | 80.4 ± 6.3 | 91.6 ± 6.0 | < 0.01 |  | 80.5 ± 6.3 | 92.1 ± 5.6 | < 0.001 |
| Fasting blood glucose (mg/dL) ^b^ | | 95 (73-206) | 100 (68-244) | < 0.001 |  | 96 (71-263) | 101 (73-208) | < 0.01 |
| Systolic BP (mmHg) | | 119.3 ± 17.2 | 126.1 ± 16.6 | < 0.01 |  | 122.1 ± 18.3 | 128.1 ± 16.0 | < 0.01 |
| Diastolic BP (mmHg) | | 71.5 ± 10.9 | 77.5 ± 10.7 | < 0.01 |  | 71.4 ± 11.1 | 75.7 ± 10.3 | < 0.01 |
| eGFR (ml/min/1.73m^2^) | | 74.6 ± 13.4 | 71.5 ± 13.8 | < 0.01 |  | 72.8 ± 13.4 | 69.6 ± 12.7 | < 0.05 |
| LDL-C (mg/dL) | | 122.4 ± 27.3 | 129.7 ± 27.9 | < 0.01 |  | 123.8 ± 27.5 | 123.2 ± 27.6 | 0.861 |
| HDL-C (mg/dL) | | 69.1 ± 16.8 | 59.4 ± 15.0 | < 0.01 |  | 71.5 ± 16.4 | 61.3 ± 15.3 | < 0.001 |
| TG (mg/dL) ^b^ | | 85 (26-921) | 112 (42-508) | < 0.001 |  | 87 (31-321) | 101 (34-380) | < 0.01 |
| AST (IU/L) | | 24.2 ± 7.5 | 27.4 ± 13.8 | < 0.01 |  | 24.4 ± 7.7 | 26.0 ± 9.1 | 0.116 |
| ALT (IU/L) | | 22.2 ± 11.0 | 30.1 ± 18.5 | < 0.01 |  | 21.8 ± 11.1 | 25.6 ± 11.6 | < 0.01 |
| GGT (IU/L) ^b^ | | 22 (6-302) | 31 (11-657) | < 0.001 |  | 22 (7-259) | 31 (11-147) | < 0.001 |
| Diabetes (%) ^a^ | | 67 (11.9) | 42 (23.7) | < 0.001 |  | 34 (10.8) | 17 (21.5) | < 0.05 |
| Hypertension (%) ^a^ | | 203 (36.1) | 91 (51.4) | < 0.001 |  | 116 (36.9) | 49 (62.0) | < 0.001 |
| Dyslipidemia (%) ^a^ | | 287 (51.0) | 115 (65.0) | < 0.001 |  | 138 (43.9) | 44 (55.7) | 0.077 |
| NAFLD (%) ^a^ | | 55 (12.0) | 64 (47.8) | < 0.001 |  | 33 (10.5) | 30 (38.0) | < 0.001 |
| Ever smoking (%) ^a^ | | 224 (39.8) | 80 (45.2) | 0.220 |  | 111 (35.4) | 34 (43.0) | 0.240 |
| Habitual alcohol intake (%) ^a^ | | 85 (15.1) | 38 (21.5) | 0.063 |  | 20 (6.4) | 10 (12.7) | 0.093 |
| Hepatitis B or C virus positive (%) ^a^ | | 22 (3.9) | 5 (2.8) | 0.648 |  | 17 (5.4) | 6 (7.6) | 0.430 |
| *PNPLA3* ^a^ | C/C | 149 (26.5) | 53 (29.9) | 0.605 |  | 85 (27.1) | 22 (27.8) | 0.931 |
|  | C/G | 305 (54.2) | 94 (53.1) |  |  | 174 (55.4) | 42 (53.2) |  |
|  | G/G | 109 (19.4) | 30 (16.9) |  |  | 55 (17.5) | 15 (19.0) |  |

The data are the means±standard deviation, median (range) for skewed variables, or the numbers of subjects (%) for categorical variables.

^a^ Fisher’s exact test. ^b^ Mann–Whitney U test (otherwise, Student’s t-test was used).

BMI, body mass index; BP, blood pressure; eGFR, estimated glomerular filtration rate; LDL-C, low-density lipoprotein cholesterol; HDL-C, high-density lipoprotein cholesterol; TG, triglyceride; AST, aspartate aminotransferase; ALT, alanine aminotransferase; GGT, gamma-glutamyl transferase; NAFLD, non-alcoholic fatty liver disease; PNPLA3, patatin-like phospholipase 3.
