# Supplementary material for: Influence of the PNPLA3 rs738409 Polymorphism on Non-Alcoholic Fatty Liver Disease and Renal Function among Normal Weight Subjects
Source: PLoS One. 2015 Jul 22;10(7):e0132640. doi: 10.1371/journal.pone.0132640 (PMC4511733; doi:10.1371/journal.pone.0132640)
Supplement: S2 Table — (DOCX) [file pone.0132640.s002.docx]

**S2 Table. Clinical characteristics of all 740 subjects stratified by weight status and the *PNPLA3* genotype.**

|  | Normal weight | | | |  | Overweight | | | |
| --- | --- | --- | --- | --- | --- | --- | --- | --- | --- |
| *PNPLA3* genotype | C/C  (N = 149) | C/G  (N = 305) | G/G  (N = 109) | *P* |  | C/C  (N = 53) | C/G  (N = 94) | G/G  (N = 30) | *P* |
| Female (%) ^a^ | 51 (34.2) | 119 (39.1) | 44 (40.4) | 0.528 |  | 15 (28.3) | 23 (24.5) | 10 (33.3) | 0.445 |
| Age (years) | 62.3 ± 10.1 | 61.9 ± 10.7 | 60.1 ± 9.8 | 0.210 |  | 60.4 ± 10.2 | 59.3 ± 11.2 | 59.2 ± 11.0 | 0.814 |
| BMI (kg/m^2^) | 21.9 ± 2.1 | 21.9 ± 2.0 | 21.7 ± 1.9 | 0.678 |  | 26.9 ± 2.2 | 26.7 ± 1.5 | 27.7 ± 3.2 | 0.082 |
| Waist circumstance (cm) | 79.8 ± 6.0 | 80.8 ± 6.5 | 80.3 ± 5.9 | 0.365 |  | 90.7 ± 5.2 | 91.4 ± 5.9 | 93.7 ± 7.2 | 0.103 |
| Fasting blood glucose (mg/dL) ^b^ | 95 (74-161) | 96 (74-206) | 94 (73-201) | 0.261 |  | 97 (81-196) | 101 (68-244) | 104 (88-168) | 0.519 |
| Systolic BP (mmHg) | 119.0 ± 16.1 | 119.5 ± 17.5 | 119.3 ± 17.7 | 0.964 |  | 125.9 ± 17.6 | 126.9 ± 16.2 | 124.1 ± 16.3 | 0.717 |
| Diastolic BP (mmHg) | 71.0 ± 11.2 | 71.4 ± 10.9 | 72.3 ± 10.6 | 0.648 |  | 77.6 ± 11.1 | 77.5 ± 10.5 | 77.2 ± 11.1 | 0.984 |
| eGFR (ml/min/1.73m^2^) | 75.7 ± 13.9 | 74.4 ± 13.2 | 73.8 ± 13.2 | 0.490 |  | 69.9 ± 13.7 | 72.1 ± 14.8 | 72.6 ± 10.5 | 0.573 |
| LDL-C (mg/dL) | 121.5 ± 25.9 | 124.0 ± 27.7 | 119.0 ± 28.0 | 0.249 |  | 126.6 ± 31.7 | 128.3 ± 25.6 | 139.2 ± 26.3 | 0.105 |
| HDL-C (mg/dL) | 68.8 ± 16.9 | 68.8 ± 17.2 | 70.4 ± 15.4 | 0.680 |  | 59.6 ± 16.0 | 58.9 ± 14.4 | 60.5 ± 15.3 | 0.876 |
| TG (mg/dL) ^b^ | 80 (30-476) | 88 (30-921) | 88 (26-520) | 0.142 |  | 111 (43-262) | 108 (42-508) | 124.5 (53-393) | 0.841 |
| AST (IU/L) | 24.1 ± 8.3 | 24.0 ± 6.9 | 24.8 ± 8.2 | 0.576 |  | 23.8 ± 4.5 | 28.6 ± 15.3 | 30.2 ± 18.2 | 0.061 |
| ALT (IU/L) | 22.0 ± 10.3 | 21.9 ± 10.5 | 23.5 ± 13.2 | 0.416 |  | 24.3 ± 8.4 | 31.1 ± 16.2 | 36.9 ± 31.3 | < 0.05 |
| GGT (IU/L) ^b^ | 23 (8-276) | 22 (6-274) | 23 (10-302) | 0.787 |  | 32 (11-168) | 31 (11-657) | 30 (15-442) | 0.915 |
| Diabetes (%) ^a^ | 20 (13.4) | 37 (12.1) | 10 (9.2) | 0.589 |  | 12 (22.6) | 23 (24.5) | 7 (23.3) | 0.971 |
| Hypertension (%) ^a^ | 55 (36.9) | 112 (36.7) | 36 (33.0) | 0.774 |  | 29 (54.7) | 49 (52.1) | 13 (43.3) | 0.635 |
| Dyslipidemia (%) ^a^ | 81 (54.4) | 159 (52.1) | 47 (43.1) | 0.170 |  | 36 (67.9) | 55 (58.5) | 24 (80.0) | 0.091 |
| NAFLD (%) ^a^ | 9 (7.2) | 34 (13.7) | 12 (14.5) | 0.130 |  | 14 (34.1) | 35 (50.7) | 15 (62.5) | 0.069 |
| Ever smoking (%) ^a^ | 53 (35.6) | 125 (41.0) | 46 (42.2) | 0.457 |  | 26 (49.1) | 43 (45.7) | 11 (36.7) | 0.531 |
| Habitual alcohol intake (%) ^a^ | 17 (11.5) | 48 (15.7) | 20 (18.7) | 0.281 |  | 11 (20.8) | 21 (22.3) | 6 (20.0) | 0.999 |
| Hepatitis B or C virus positive (%) ^a^ | 7 (4.7) | 8 (2.6) | 7 (6.4) | 0.170 |  | 1 (1.9) | 4 (4.3) | 0 (0.0) | 0.577 |

The data are the means±standard deviation, median (range) for skewed variables, or the numbers of subjects (%) for categorical variables.

^a^ Fisher’s exact test. ^b^ Kruskal-Wallis test (otherwise, one-way ANOVA was used).

PNPLA3, patatin-like phospholipase 3; BMI, body mass index; BP, blood pressure; eGFR, estimated glomerular filtration rate; LDL-C, low-density lipoprotein cholesterol; HDL-C, high-density lipoprotein cholesterol; TG, triglyceride; AST, aspartate aminotransferase; ALT, alanine aminotransferase; GGT, gamma-glutamyl transferase; ; NAFLD, non-alcoholic fatty liver disease.
