# Supplementary material for: Influence of the PNPLA3 rs738409 Polymorphism on Non-Alcoholic Fatty Liver Disease and Renal Function among Normal Weight Subjects
Source: PLoS One. 2015 Jul 22;10(7):e0132640. doi: 10.1371/journal.pone.0132640 (PMC4511733; doi:10.1371/journal.pone.0132640)
Supplement: S3 Table — (DOCX) [file pone.0132640.s003.docx]

**S3 Table. Clinical characteristics at baseline of 393 subjects included in the longitudinal analyses stratified by the weight status and the *PNPLA3* genotype.**

|  | Normal weight | | | |  | Overweight | | | |
| --- | --- | --- | --- | --- | --- | --- | --- | --- | --- |
| *PNPLA3* genotype | C/C  (N = 85) | C/G  (N = 174) | G/G  (N = 55) | *P* |  | C/C  (N = 22) | C/G  (N = 42) | G/G  (N = 15) | *P* |
| Female (%) ^a^ | 30 (35.3) | 77 (44.3) | 24 (43.6) | 0.383 |  | 8 (36.4) | 10 (23.8) | 7 (46.7) | 0.220 |
| Age (years) | 67.3 ± 5.6 | 67.9 ± 5.9 | 66.6 ± 5.9 | 0.358 |  | 67.0 ± 6.6 | 67.6 ± 6.7 | 66.9 ± 5.7 | 0.908 |
| BMI (kg/m^2^) | 21.7 ± 2.0 | 21.7 ± 2.0 | 21.9 ± 2.0 | 0.895 |  | 26.7 ± 2.3 | 26.7 ± 1.8 | 27.0 ± 2.3 | 0.875 |
| Waist circumstance (cm) | 79.9 ± 5.9 | 80.7 ± 6.8 | 80.9 ± 5.2 | 0.626 |  | 90.6 ± 5.7 | 92.2 ± 5.4 | 93.3 ± 6.0 | 0.437 |
| Fasting blood glucose (mg/dL) ^b^ | 95 (71-263) | 97 (74-230) | 96 (78-121) | 0.589 |  | 100.5 (85-138) | 103 (73-208) | 97 (85-119) | 0.356 |
| Systolic BP (mmHg) | 120.7 ± 17.2 | 122.9 ± 19.0 | 121.7 ± 17.9 | 0.639 |  | 127.9 ± 17.6 | 128.0 ± 16.3 | 128.9 ± 13.4 | 0.981 |
| Diastolic BP (mmHg) | 70.7 ± 11.9 | 71.8 ± 11.0 | 71.6 ± 10.2 | 0.767 |  | 76.0 ± 11.4 | 75.6 ± 10.4 | 75.5 ± 9.2 | 0.985 |
| eGFR (ml/min/1.73m^2^) | 74.2 ± 13.8 | 72.5 ± 13.2 | 71.5 ± 13.6 | 0.550 |  | 68.1 ± 11.8 | 70.8 ± 14.1 | 68.4 ± 10.3 | 0.745 |
| LDL-C (mg/dL) | 123.8 ± 26.4 | 125.6 ± 28.3 | 118.3 ± 26.0 | 0.231 |  | 111.2 ± 30.3 | 123.4 ± 24.5 | 140.3 ± 23.9 | 0.006 |
| HDL-C (mg/dL) | 70.8 ± 16.9 | 71.7 ± 16.5 | 72.2 ± 15.6 | 0.882 |  | 63.6 ± 19.0 | 60.3 ± 13.4 | 60.7 ± 15.1 | 0.711 |
| TG (mg/dL) ^b^ | 81 (34-310) | 88 (36-321) | 89 (31-264) | 0.754 |  | 105.5 (43-380) | 106 (34-299) | 95 (62-134) | 0.550 |
| AST (IU/L) | 24.1 ± 6.3 | 24.0 ± 7.4 | 26.1 ± 10.0 | 0.188 |  | 23.5 ± 4.2 | 28.1 ± 11.4 | 23.8 ± 4.7 | 0.092 |
| ALT (IU/L) | 21.4 ± 8.4 | 21.2 ± 10.3 | 24.2 ± 15.8 | 0.206 |  | 22.3 ± 6.0 | 28.0 ± 14.2 | 23.5 ± 7.4 | 0.123 |
| GGT (IU/L) ^b^ | 21 (10-141) | 21 (7-259) | 25 (8-174) | < 0.05 |  | 35 (13-116) | 32 (11-147) | 25 (14-63) | 0.099 |
| Diabetes (%) ^a^ | 10 (11.8) | 20 (11.5) | 4 (7.3) | 0.704 |  | 5 (22.7) | 9 (21.4) | 3 (20.0) | 1.000 |
| Hypertension (%) ^a^ | 28 (32.9) | 70 (40.2) | 18 (32.7) | 0.420 |  | 14 (63.6) | 26 (61.9) | 9 (60.0) | 1.000 |
| Dyslipidemia (%) ^a^ | 35 (41.2) | 83 (47.7) | 20 (36.4) | 0.279 |  | 14 (63.6) | 21 (50.0) | 9 (60.0) | 0.566 |
| NAFLD (%) ^a^ | 2 (2.7) | 22 (13.9) | 5 (11.4) | < 0.05 |  | 7 (35.0) | 12 (41.4) | 3 (20.0) | 0.365 |
| Ever smoking (%) ^a^ | 33 (38.8) | 61 (35.1) | 17 (30.9) | 0.638 |  | 9 (40.9) | 21 (50.0) | 4 (26.7) | 0.286 |
| Habitual alcohol intake (%) ^a^ | 6 (7.1) | 9 (5.2) | 5 (9.1) | 0.510 |  | 1 (4.5) | 9 (21.4) | 0 (0.0) | 0.058 |
| Hepatitis B or C virus positive (%) ^a^ | 4 (4.7) | 7 (4.0) | 6 (10.9) | 0.156 |  | 2 (9.1) | 4 (9.5) | 0 (0.0) | 0.629 |

The data are the means±standard deviation, median (range) for skewed variables, or the numbers of subjects (%) for categorical variables.

^a^ Fisher’s exact test. ^b^ Kruskal-Wallis test (otherwise, one-way ANOVA was used).

PNPLA3, patatin-like phospholipase 3; BMI, body mass index; BP, blood pressure; eGFR, estimated glomerular filtration rate; LDL-C, low-density lipoprotein cholesterol; HDL-C, high-density lipoprotein cholesterol; TG, triglyceride; AST, aspartate aminotransferase; ALT, alanine aminotransferase; GGT, gamma-glutamyl transferase; ; NAFLD, non-alcoholic fatty liver disease.
