# Supplementary material for: Influence of the PNPLA3 rs738409 Polymorphism on Non-Alcoholic Fatty Liver Disease and Renal Function among Normal Weight Subjects
Source: PLoS One. 2015 Jul 22;10(7):e0132640. doi: 10.1371/journal.pone.0132640 (PMC4511733; doi:10.1371/journal.pone.0132640)
Supplement: S4 Table — (DOCX) [file pone.0132640.s004.docx]

**S4 Table. The effect of the *PNPLA3* genotype on the risk of NAFLD identified in the cross-sectional and longitudinal bi-variable logistic regression analyses.**

|  | | | All | |  | Normal weight | |  | Overweight | |
| --- | --- | --- | --- | --- | --- | --- | --- | --- | --- | --- |
|  |  |  | NAFLD /  Non-NAFLD^a^ | OR (95% CI) ^b^ |  | NAFLD /  Non-NAFLD^a^ | OR (95% CI) ^b^ |  | NAFLD /  Non-NAFLD^a^ | OR (95% CI) ^b^ |
| Cross-sectional analysis | | |  |  |  |  |  |  |  |  |
| *PNPLA3* | C/C | | 23 / 143 | 1 |  | 9 / 116 | 1 |  | 14 / 27 | 1 |
|  | C/G | | 69 / 249 | 1.72 (1.03-2.88) |  | 34 / 215 | 2.04 (0.95-4.40) |  | 35 / 34 | 1.99 (0.89-4.42) |
|  | G/G | | 27 / 80 | 2.10 (1.13-3.90) |  | 12 / 71 | 2.18 (0.87-5.43) |  | 15 / 9 | 3.21 (1.13-9.17) |
| Age | | |  | 0.98 (0.96-1.00) |  | - | 0.99 (0.97-1.02) |  | - | 0.97 (0.95-1.00) |
| Gender | | Male | 83 / 267 | 1 |  | 36 / 223 | 1 |  | 47 / 44 | 1 |
|  | | Female | 36 / 205 | 0.57 (0.37-0.87) |  | 19 / 179 | 0.66 (0.37-1.19) |  | 17 / 26 | 0.61 (0.29-1.28) |
| BMI | | |  | 1.55 (1.41-1.71) |  | - | 1.83 (1.48-2.25) |  | - | 1.34 (1.09-1.64) |
| Diabetes | | Absent | 91 / 421 | 1 |  | 44 / 362 | 1 |  | 47 / 59 | 1 |
|  | | Present | 28 / 51 | 2.54 (1.52-4.25) |  | 11 / 40 | 2.26 (1.08-4.73) |  | 17 / 11 | 1.94 (0.83-4.54) |
| Hypertension | | Absent | 64 / 297 | 1 |  | 32 / 262 | 1 |  | 32 / 35 | 1 |
|  | | Present | 55 / 175 | 1.46 (0.97-2.19) |  | 23 / 140 | 1.35 (0.76-2.39) |  | 32 / 35 | 1.00 (0.51-1.97) |
| Dyslipidemia | | Absent | 23 / 237 | 1 |  | 8 / 207 | 1 |  | 15 / 30 | 1 |
|  | | Present | 96 / 235 | 4.21 (2.58-6.87) |  | 47 195 | 6.24 (2.87-13.53) |  | 49 / 40 | 2.45 (1.16-5.17) |
|  | | |  |  |  |  |  |  |  |  |
| Longitudinal analysis | | |  |  |  |  |  |  |  |  |
| *PNPLA3* | C/C | | 15 / 80 | 1 |  | 7 / 68 | 1 |  | 8 / 12 | 1 |
|  | C/G | | 42 / 145 | 1.92 (1.30-5.49) |  | 28 / 129 | 3.30 (1.20-9.06) |  | 14 / 16 | 1.86 (0.71-4.88) |
|  | G/G | | 18 / 41 | 2.61 (1.16-5.88) |  | 11 / 34 | 4.20 (1.34-13.16) |  | 7 / 7 | 1.90 (0.62-5.81) |
| Age | | | - | 0.97 (0.93-1.02) |  | - | 0.97 (0.91-1.04) |  | - | 0.98 (0.92-1.05) |
| Gender | | Male | 48 / 148 | 1 |  | 27 /128 | 1 |  | 21 / 20 | 1 |
|  | | Female | 27 / 118 | 1.07 (0.62-1.83) |  | 19 / 103 | 1.28 (0.66-2.49) |  | 8 / 15 | 1.05 (0.44-2.48) |
| BMI | | | - | 1.48 (1.35-1.62) |  | - | 1.92 (1.51-2.43) |  | - | 1.29 (1.11-1.48) |
| Diabetes | | Absent | 50 / 225 | 1 |  | 36 / 197 | 1 |  | 14 / 28 | 1 |
|  | | Present | 25 / 41 | 2.73 (1.58-4.73) |  | 10 / 34 | 2.28 (1.07-4.88) |  | 15 / 7 | 2.09 (0.98-4.46) |
| Hypertension | | Absent | 34 / 129 | 1 |  | 24 / 123 | 1 |  | 10 / 6 | 1 |
|  | | Present | 41 / 137 | 1.29 (0.81-2.06) |  | 22 / 108 | 1.19 (0.66-2.13) |  | 19 / 29 | 0.58 (0.27-1.23) |
| Dyslipidemia | | Absent | 22 / 126 | 1 |  | 12 / 111 | 1 |  | 10 / 15 | 1 |
|  | | Present | 53 / 140 | 2.64 (1.65-4.21) |  | 34 / 120 | 2.37 (1.27-4.40) |  | 19 / 20 | 2.45 (1.22-4.94) |

^a^ In the longitudinal analysis, the numbers of NAFLD subjects and non-NAFLD subjects at the endpoint are shown.

^b^ Not adjusted.

PNPLA3, patatin-like phospholipase 3; NAFLD, non-alcoholic fatty liver disease; OR, odds ratio; CI, confidence interval; BMI, body mass index.
