# Supplementary material for: Influence of the PNPLA3 rs738409 Polymorphism on Non-Alcoholic Fatty Liver Disease and Renal Function among Normal Weight Subjects
Source: PLoS One. 2015 Jul 22;10(7):e0132640. doi: 10.1371/journal.pone.0132640 (PMC4511733; doi:10.1371/journal.pone.0132640)
Supplement: S5 Table — (DOCX) [file pone.0132640.s005.docx]

**S5 Table. The effect of the *PNPLA3* genotype on the values of eGFR identified in the cross-sectional and longitudinal bi-variable multiple linear regression analyses.**

|  | | All | | | |  | Normal weight | | | |  | Overweight | | | |
| --- | --- | --- | --- | --- | --- | --- | --- | --- | --- | --- | --- | --- | --- | --- | --- |
|  |  | N | B ^a^ | SE | *P* |  | N | B ^a^ | SE | *P* |  | N | B ^a^ | SE | *P* |
| Cross-sectional analysis | |  |  |  |  |  |  |  |  |  |  |  |  |  |  |
| *PNPLA3* | C/C | 202 | 0 |  |  |  | 149 | 0 |  |  |  | 53 | 0 |  |  |
|  | C/G | 399 | -0.10 | 1.15 | 0.929 |  | 305 | -0.59 | 1.31 | 0.65 |  | 94 | 1.01 | 2.35 | 0.67 |
|  | G/G | 139 | -0.22 | 1.47 | 0.880 |  | 109 | -1.59 | 1.66 | 0.34 |  | 30 | 3.88 | 3.12 | 0.22 |
| Age | | - | -0.43 | 0.04 | < 0.001 |  | - | -0.44 | 0.04 | < 0.001 |  | - | -0.46 | 0.08 | < 0.001 |
| Gender | Male | 478 | 0 |  |  |  | 349 | 0 |  |  |  | 129 | 0 |  |  |
|  | Female | 262 | 4.92 | 1.01 | < 0.001 |  | 214 | 4.07 | 1.13 | < 0.001 |  | 48 | 7.37 | 2.25 | < 0.01 |
| BMI | | - | -0.41 | 0.16 | < 0.05 |  | - | -0.79 | 0.27 | < 0.01 |  | - | 0.88 | 0.49 | 0.076 |
| Diabetes | Absent | 631 | 0 |  |  |  | 496 | 0 |  |  |  | 135 | 0 |  |  |
|  | Present | 109 | -3.20 | 1.37 | < 0.05 |  | 67 | -1.74 | 1.71 | 0.307 |  | 42 | -4.92 | 2.39 | < 0.05 |
| Hypertension | Absent | 446 | 0 |  |  |  | 360 | 0 |  |  |  | 86 | 0 |  |  |
|  | Present | 294 | -6.75 | 0.97 | < 0.001 |  | 203 | -5.66 | 1.13 | < 0.001 |  | 91 | -9.38 | 1.93 | < 0.001 |
| Dyslipidemia | Absent | 338 | 0 |  |  |  | 276 | 0 |  |  |  | 62 | 0 |  |  |
|  | Present | 402 | -3.88 | 0.97 | < 0.001 |  | 287 | -4.84 | 1.09 | < 0.001 |  | 115 | 0.31 | 2.16 | 0.886 |
| FLD | Absent | 590 | 0 |  |  |  | 496 | 0 |  |  |  | 94 | 0 |  |  |
|  | Present | 150 | -2.09 | 1.21 | 0.085 |  | 67 | -2.81 | 1.71 | 0.100 |  | 83 | 0.55 | 2.06 | 0.791 |
|  | |  |  |  |  |  |  |  |  |  |  |  |  |  |  |
| Longitudinal analysis | |  |  |  |  |  |  |  |  |  |  |  |  |  |  |
| *PNPLA3* | C/C | 107 | 0 |  |  |  | 85 | 0 |  |  |  | 22 | 0 |  |  |
|  | C/G | 216 | -0.61 | 1.39 | 0.660 |  | 174 | -1.59 | 1.55 | 0.305 |  | 42 | 2.98 | 2.76 | 0.279 |
|  | G/G | 70 | -2.45 | 1.73 | 0.158 |  | 55 | -3.32 | 1.97 | 0.091 |  | 15 | 0.91 | 3.32 | 0.784 |
| Age | | - | -0.45 | 0.86 | < 0.001 |  | - | -0.49 | 0.10 | <0.001 |  | - | -0.32 | 0.16 | < 0.05 |
| Gender | Male | 237 | 0 |  |  |  | 183 | 0 |  |  |  | 54 | 0 |  |  |
|  | Female | 156 | 4.95 | 1.22 | < 0.001 |  | 131 | 5.09 | 1.35 | < 0.001 |  | 25 | 2.76 | 3.01 | 0.359 |
| BMI | |  |  |  |  |  | - |  |  |  |  | - | 0.26 | 0.53 | 0.627 |
| Diabetes | Absent | 342 | 0 |  |  |  | 280 | 0 |  |  |  | 62 | 0 |  |  |
|  | Present | 51 | 0.69 | 1.48 | 0.642 |  | 34 | 0.32 | 1.92 |  |  | 17 | 3.73 | 2.28 | 0.102 |
| Hypertension | Absent | 228 | 0 |  |  |  | 198 | 0 |  |  |  | 30 | 0 |  |  |
|  | Present | 165 | -4.03 | 1.08 | < 0.001 |  | 116 | -3.36 | 1.25 | < 0.01 |  | 49 | -4.84 | 2.28 | < 0.05 |
| Dyslipidemia | Absent | 211 | 0 |  |  |  | 176 | 0 |  |  |  | 35 | 0 |  |  |
|  | Present | 182 | -3.35 | 1.03 | < 0.01 |  | 138 | -3.31 | 1.16 | < 0.01 |  | 44 | -2.87 | 2.13 | 0.177 |
| FLD | Absent | 330 | 0 |  |  |  | 281 | 0 |  |  |  | 49 | 0 |  |  |
|  | Present | 63 | -3.72 | 1.48 | < 0.05 |  | 33 | -2.36 | 2.12 | 0.265 |  | 30 | -3.77 | 2.04 | 0.064 |

^a^ Adjusted by all covariates.

PNPLA3, patatin-like phospholipase 3; eGFR, estimated glomerular filtration rate; B, partial regression coefficient; SE, standard error; BMI, body mass index; FLD, fatty liver disease.
